# No Cases of PANDAS on Follow-Up of Patients Referred to a Pediatric Movement Disorders Clinic

**DOI:** 10.3389/fped.2014.00104

**Published:** 2014-09-25

**Authors:** Sarah Kilbertus, Renee Brannan, Erick Sell, Asif Doja

**Affiliations:** ^1^Faculty of Medicine, University of Ottawa, Ottawa, ON, Canada; ^2^Department of Neurology, Children’s Hospital of Eastern Ontario (CHEO), Ottawa, ON, Canada

**Keywords:** PANDAS, streptococcal infection, OCD, Tourette’s syndrome, tics

## Abstract

**Introduction:** Pediatric autoimmune neuropsychiatric disorders associated with streptococcal infection (PANDAS) remains a controversial diagnosis and it is unclear how frequently it is encountered in clinical practice. Our study aimed to determine how many children with acute-onset tics and/or Obsessive–Compulsive Disorder (OCD) met criteria for PANDAS.

**Materials and methods:** A retrospective review was performed on 39 children who presented to a movement disorders clinic with acute-onset tics or OCD from 2005 to 2012.

**Results:** Out of 284 patients seen over the course of 7 years, only 39 had acute-onset tics and/or OCD symptoms. None of the 39 children who presented to us acutely met full criteria for PANDAS. Thirty-eight percent had no association between their symptoms and group A beta-hemolytic streptococcal infection, while 54% had prior inconclusive laboratory testing done and no exacerbations during the course of the study. Only 8% of patients had an acute exacerbation after their initial visit; however, testing for GAHBS in these patients was negative

**Discussion:** Our results support the notion that PANDAS, if it exists, is an exceedingly rare diagnosis encountered in a pediatric movement disorder clinic. While none of our patients met criteria for PANDAS, two with acute-onset OCD would have met criteria for pediatric acute-onset neuropsychiatric syndrome (PANS) indicating that PANS may be a more appropriate diagnosis.

## Introduction

The diagnosis of pediatric autoimmune neuropsychiatric disorders associated with streptococcal infection (PANDAS) has been controversial since its initial description. Researchers originally described a subgroup of patients who developed obsessive–compulsive disorder (OCD)-like behaviors and/or tics following a group A beta-hemolytic Streptococcal (GABHS) infection (1). The current diagnostic criteria for PANDAS are as follows: (1) the presence of OCD or tic disorder; (2) prepubertal age at onset (usually ages 3–12 years); (3) abrupt symptom onset and/or episodic course of symptom severity; (4) temporal association between symptom exacerbations and GABHS infection; and (5) association with neurologic abnormalities during symptom exacerbation (2). Currently, the exact pathophysiology of PANDAS is not fully understood. It is postulated that in these patients, a GABHS infection results in the formation of anti-streptococcal antibodies that attack specific areas of the brain creating an autoimmune disease similar to Sydenham’s chorea (3).

There have been criticisms of the PANDAS hypothesis, questioning whether PANDAS is in fact a unique entity, or is simply a coincidental association between an extremely common bacterial infection and the concurrent but unrelated emergence of a neuropsychiatric disorder (4–6). In light of this controversy, our study aimed to discern how many patients who presented to a pediatric movement disorders practice with acute-onset tics, Tourette’s syndrome, or OCD met the criteria for PANDAS.

## Materials and Methods

This study was conducted at the Movement Disorders Clinic at the Children’s Hospital of Eastern Ontario (CHEO) in Ottawa, ON, Canada, a hospital with over 300,000 children ages 0–19 years in its catchment area. Most patients with new onset tics in our catchment area are referred to this clinic; additionally, the director (AD) has made other services, such as general pediatrics and psychiatry, aware of the desire to assess patients with possible PANDAS. As such, patients with tic disorders or OCD who are seen in other clinics are often referred to the Movement Disorders Clinic if the diagnosis of PANDAS is entertained. Acute symptom onset has been deemed the most useful in identifying children in the PANDAS group (5). As such, patients with acute-onset of symptoms were followed in our clinic over time. For our purposes, acute onset was defined as the parents or child being able to identify the day or week the symptoms began.

Many patients referred to our clinic had throat swabs, anti-streptolysin O titers (ASOT), or anti-DNAse B performed prior to them being seen in our clinic. However, given the fact these that tests were often performed outside of our institution by a variety of practitioners, it was difficult to accurately correlate the timing of these tests with symptom exacerbation. As such, we felt that PANDAS could only be accurately diagnosed by following patients in our clinic after their initial appointment.

For the purposes of our study, “time zero” was the date when the patients initially presented to our clinic. Patients were then followed over time – families were asked to phone the clinic immediately if the patient had an exacerbation in symptoms; in these cases, a throat swab and either an ASOT or anti-DNAse B would be done immediately (within 7 days), and convalescent titers would be done 2–6 weeks later. A diagnosis of PANDAS would be made if, during the time of follow-up, a patient had one or more exacerbations in symptomatology associated with an acute GABHS infection (defined as a positive throat swab *and* a positive ASOT and/or anti-DNAse B acutely, with normal or decreasing serologic titers in the convalescent phase).

We retrospectively reviewed this data for patients who presented between 2005 and 2012 including: demographic information, date of acute onset of symptoms, association with GABHS infection including lab results for throat swabs and ASOT and/or anti-DNAse B levels, clinical symptoms, length of follow-up, and number of acute exacerbations since first neurology consultation and the patient’s final diagnosis. Patients were excluded if their final diagnosis was rheumatic fever/Sydenham’s chorea, or if the acute emergence of their tics coincided with the initiation of a stimulant medication.

This study received ethical approval from the Children’s Hospital of Eastern Ontario Research Ethics Board.

## Results

Out of 284 children (72% male, 28% female) who presented to our clinic with tics, Tourette’s syndrome, or OCD, 39 (14%) had an acute onset of symptoms and were followed in our clinic over time. The mean length of follow-up was 2 years, ranging from 0 to 5 years. Eleven patients (28%) were lost to follow-up. Thirty-one (79%) patients had tics, 3 (8%) had OCD, and 5 (13%) had tics and OCD.

Prior to being seen at our clinic, 24 patients had prior laboratory investigations done (10 had ASOT, throat swab and an anti-DNse B, 8 had throat swabs only, 3 had ASOT and an anti-DNAse B, 2 had ASOT and a throat swab, and 1 had ASOT only) in an attempt to detect a GABHS infection, while 15 patients had no laboratory investigations done indicating that PANDAS was not considered as a possible diagnosis for their symptomatology. Out of those 24 patients, 17 had at least one positive investigation, and 7 had normal results. However all of these investigations were undertaken by other providers (and consequently it was difficult to establish the timing of the test with regards to the exacerbation) or they were not taken during an acute exacerbation at all and thus a diagnosis of PANDAS could not be made. Upon following each patient after their initial consultation in our clinic, only three patients reported acute exacerbations of tics and/or OCD (see Table 1 below). These patients had an ASOT, anti-DNAse B, and throat swab done within 1 week of the patient informing our clinic of an exacerbation. As is evident from the results in Table 1, none of these patients had positive throat swabs or antibody titers needed to make a PANDAS diagnosis. The final diagnoses of these patients were: 14 (35.9%) with transient tics disorder of childhood, 10 (25.6%) with tic disorder not otherwise specified, 9 (23.1%) with Tourette’s syndrome, 4 (10.3%) with OCD, and 2 (5.1%) with chronic motor tic.

**Table 1:** **Summary throat swab findings, serology results, and final diagnosis of patients who developed acute tic exacerbations after initial visit**.

Patient	Exacerbations	Throat swab	ASOT (IU/mL)	Anti-DNase B (unit/mL)	Final diagnosis
Patient A – first assessed on October 2011	Exacerbations in February 2012, December 2012, May 2013	Negative, February 2012 Negative, December 2012 Negative, May 2013	51 (low) February 2012 181 (high) December 2012 105 (low) May 2013	Not done	Tourette’s syndrome and ADHD
Patient B – first assessed on June 2007	Exacerbation April 2009	Negative April 2009	783 (high) April 6th 2009 387 (high) April 22nd 2009	240 (high) April 22nd 2009	Tourette’s syndrome
Patient C – first assessed on October 2008	Exacerbation in October 2010	Negative October 2010	47 (low) October 2010	85 (low) October 2010	Tic disorder NOS and ADHD

## Discussion

Very few patients in our study with an initial acute presentation had a subsequent acute relapse. Those who did have acute exacerbations did not have evidence for a co-existing GABHS infection; as such, no patients met criteria for PANDAS. Thus, while our study cannot “refute” the existence of PANDAS, it is reasonable to state that it would have to be considered an extremely uncommon diagnosis, given the fact that no patients were diagnosed conclusively during the 7 years of the study.

Our results mirror those by Gabbay et al., who found that when strict diagnostic criteria were applied, most children (19/31 or 61.3%) who initially received PANDAS diagnoses in the community did not meet the full criteria for PANDAS (7).

In light of the controversy surrounding PANDAS, researchers have discussed the possibility of redefining PANDAS into a broader syndrome termed childhood acute neuropsychiatric symptoms (CANS) (4) or pediatric acute-onset neuropsychiatric syndrome (PANS) (8). A diagnosis of CANS requires only the acute onset of neuropsychiatric symptoms, followed by a thorough history and physical examination and an active search for a specific etiology (4). PANS, however, requires abrupt onset of OCD or severely restricted food intake; concurrent presence of at least two neuropsychiatric symptom with similarly severe and acute onset (i.e., anxiety, emotional lability, aggression, sensory or motor abnormalities, somatic signs and symptoms); and symptoms that are not better explained by a known neurological or medical disorder (8). Both CANS and PANS are much broader than PANDAS and include not only disorders potentially associated with a preceding infection, but also acute-onset neuropsychiatric disorders without an obvious precipitant (8). While none of our patients met criteria for PANDAS, two with acute-onset OCD would have met criteria for PANS, indicating that there may be a stronger basis for the existence of PANS as an entity or, at the very least, that PANS is more common than PANDAS.

There are several limitations, which need to be taken into account when interpreting our results. There is the possibility that certain PANDAS cases were referred to other physicians in or outside of our institution and were therefore missed; however, we did make it known to other providers that we were interested in assessing patients with possible PANDAS. Additionally, 11 patients were lost to follow-up thus we are unable to accurately determine in those patients whether or not they had acute exacerbations, which would fulfill the PANDAS criteria.

Another limitation is that we did not assess patients during their first acute presentation. More often, these patients were seen initially by their primary care practitioner or by emergency room clinicians. Thus, many patients had laboratory tests performed prior to them being seen in our clinic. Since we were not able to accurately correlate the timing of the tests with the acute clinical presentation, some genuine GABHS-associated tic or OCD exacerbations that would have met criteria for PANDAS may have been missed. However, given that PANDAS patients typically have a relapsing course (2), one would assume that most of these patients should have gone on to have subsequent exacerbations of their symptoms associated with GABHS, which we did not see in our study population.

It is clear that further prospective studies are needed in order to elucidate whether or not PANDAS is a true clinical entity; our results suggest that in a tertiary movement disorders clinic CANS or PANS would be better diagnostic categories for pediatric patients who present with acute-onset neuropsychiatric symptoms.

## Author Contributions

Sarah Kilbertus was involved in the conception and design of the work, the analysis, the interpretation of results, and the drafting of the work. Renee Brannan was involved in the conception and design of the work, data analysis, and revising the draft for important intellectual content. Erick Sell was involved in the interpretation of data from the work and in revising the draft for important intellectual content. Asif Doja was involved in the conception and design of the work, the analysis, interpretation of results, and in revising the draft for important intellectual content. All authors approved the final version of the manuscript.

## Conflict of Interest Statement

The authors declare that the research was conducted in the absence of any commercial or financial relationships that could be construed as a potential conflict of interest.
